# Technical considerations and outcomes for ileal ureter replacement: a retrospective study in China

**DOI:** 10.1186/s12893-019-0472-1

**Published:** 2019-01-18

**Authors:** Wenlong Zhong, Peng Hong, Guangpu Ding, Kunlin Yang, Xuesong Li, Junsheng Bao, Guochang Bao, Liang Cui, Changping Men, Zhe Li, Peng Zhang, Ning Chu, Liqun Zhou

**Affiliations:** 1 Department of Urology, Peking University First Hospital, Institute of Urology, National Urological Cancer Center, Peking University, No.8 Xishiku St, Xicheng District, Beijing, 100034 China; 2 Department of Urology, Lanzhou University Second Hospital, Lanzhou, Gansu China; 3 Department of Urology, Affiliated Hospital of Chifeng University, Chifeng, Inner Mongolia China; 4 Department of Urology, The General Hospital of Civil Aviation Administration of China, Beijing, China; 5 Department of Urology, Qindao University Medical College Affiliated Yantai Yuhuangding Hospital, Yantai, Shandong China; 6 Department of Urology, China Meitan General Hospital, Beijing, China; 7 Department of Urology, Heilongjiang provincial hospital, Harbin, Heilongjiang China

**Keywords:** Ileum, Ureter replacement, Reconstructive surgical procedures, Outcome

## Abstract

**Background:**

Ileal ureter replacement is an alternative treatment for various length ureter defects. We present our experience and outcome of ileal ureter replacement in China.

**Methods:**

We retrospectively collected data of patients who underwent ileal ureter replacement between January 2010 and January 2015. We reviewed the medical history, indications for surgery, operative data, perioperative data, and outcomes. Besides, follow-up data included symptom, urine routine test, serum creatinine, serum electrolyte status, and radiographic test.

**Results:**

There were 23 patients who underwent ileal ureter replacement by the same surgeon. Twenty patients were performed unilateral ileal ureter replacement, two patients underwent a combination of ileal ureter replacement and Boari flap-psoas hitch, and one received bilateral ileal ureter replacement. Among these patients, the main cause leading to surgical treatment was iatrogenic injuries (*n* = 15), especially urinary surgery procedure (*n* = 11). The median follow-up time was 45 months. There were 6 early complications and 6 late complications after operation. Only one patient suffered from small bowel-related complication and was cured by conservative treatment. Only the patient who underwent bilateral ileal ureter replacement had metabolic acidosis. And 22 patients (95.7%) had a good renal function.

**Conclusions:**

Ileal ureter replacement is an efficacious and safe procedure for the therapy of long ureteral defects. With appropriate technical considerations, the complication rate may decrease.

**Electronic supplementary material:**

The online version of this article (10.1186/s12893-019-0472-1) contains supplementary material, which is available to authorized users.

## Background

Long segment ureteral defect, occasionally requiring a definitive reconstructive procedure, presents a complex challenge to urologists. Depending on the length and position of the ureteral injury, ureteroureterostomy, psoas hitch, Boari flap, and autotransplantation can be used as alternative techniques for treatment [1]. However, all of these techniques have inherent limitations owing to the restricted availability of the ureter or bladder. Ileal ureter replacement is occasionally considered as the last resort for more extensive defects that are not amenable to reconstruction by other means.

Ileal ureter replacement was introduced by Shoemaker [2] in 1906 to treat long segment ureteral defect and was popularized by Goodwin et al. [3] in 1959. Studies were subsequently conducted to evaluate different alternative materials for the ureter; meanwhile, an increasing number of cases that underwent ileal ureteral substitution were reported in published studies [4–8]. Currently, the use of ileal segment for ureteral replacement has become a valuable alternative in reconstructive urology.

However, technical difficulties and surgical complications limit the extended use of the procedure. To our knowledge, published studies on the use of this procedure in the Asian population remain limited. In the present study, we describe our technical considerations in ileal ureter replacement and present a retrospective experience among patients with long ureteral injuries in China.

## Methods

### Patients

A retrospective study of ileal ureter replacement in patients with long ureteral defects was conducted from January 2010 to January 2015. Preoperative radiographic examinations, such as antegrade and retrograde pyelography, nuclear renography, computed tomography urography (CTU), and magnetic resonance urography (MRU) were used to evaluate the defects. Data on patient characteristics, indications for surgery, intraoperative variables, surgical complications, and postoperative outcomes were collected. The present study was approved by Peking University First Hospital ethics committee. Written, informed consent was obtained from all individual participants in the study.

### Surgical technique

**Fig. 1:**
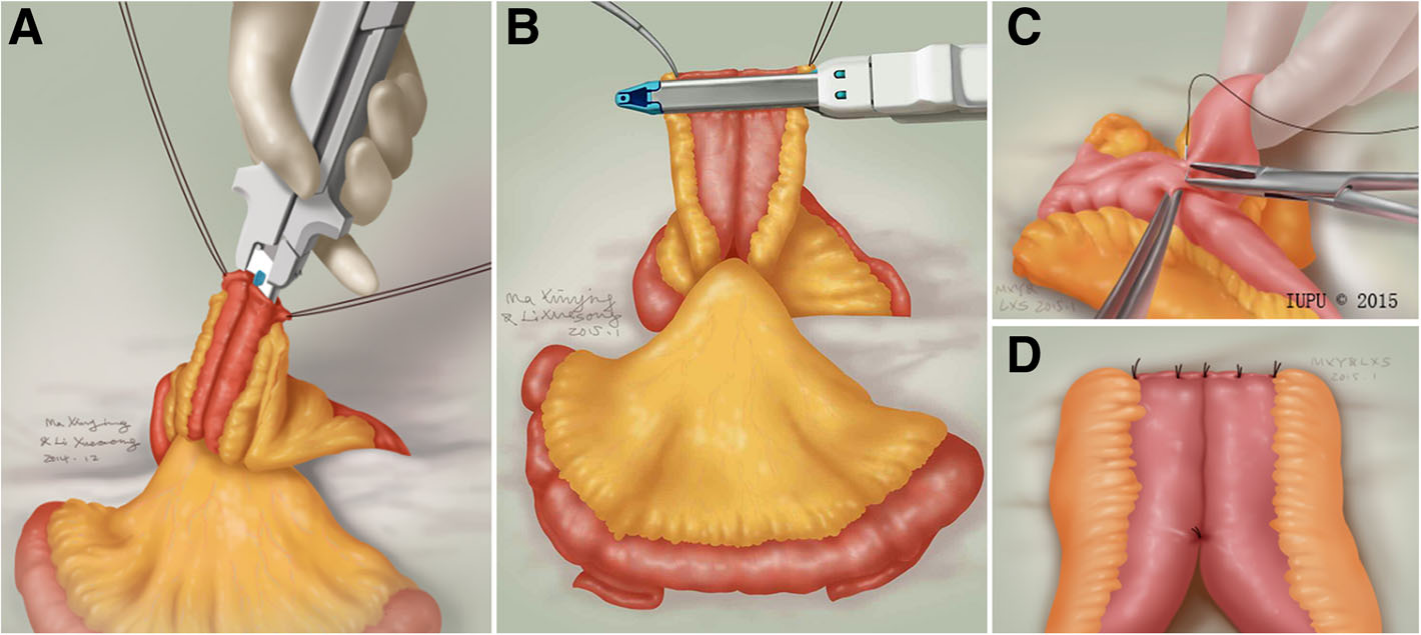
Our modified stapled side-to-side anastomosis. **a** Side-to-side anastomosis at the anti-mesenteric edge, completed using a linear stapler through the vertical incision, **b**. Open ends of the proximal and distal ilea, closed using another linear stapler load, **c** and **d**. The anastomotic edge was strengthened by interrupted Lembert sutures

The procedure for ileal ureter replacement was similar to that described in a previous study [9]; however, several minor surgical steps were used during our procedure. Ureteral exposure was achieved with mid-line incision. After the involved ureteral segment was dissected, the renal pelvis or ureter proximal to the injury site was widely spatulated for subsequent anastomosis. An appropriate ileal segment was divided 25–30 cm proximal from the ileocecal junction after measuring the length of the defect. The isolated ileal segment was used to bridge the defect in an isoperistaltic direction. Bowel continuity was restored with stapled side-to-side anastomosis using two linear staplers (Fig. 1, a and b) and interrupted Lembert sutures were subsequently used to strengthen the anastomotic edge (Fig. 1, c and d).

**Fig. 2:**
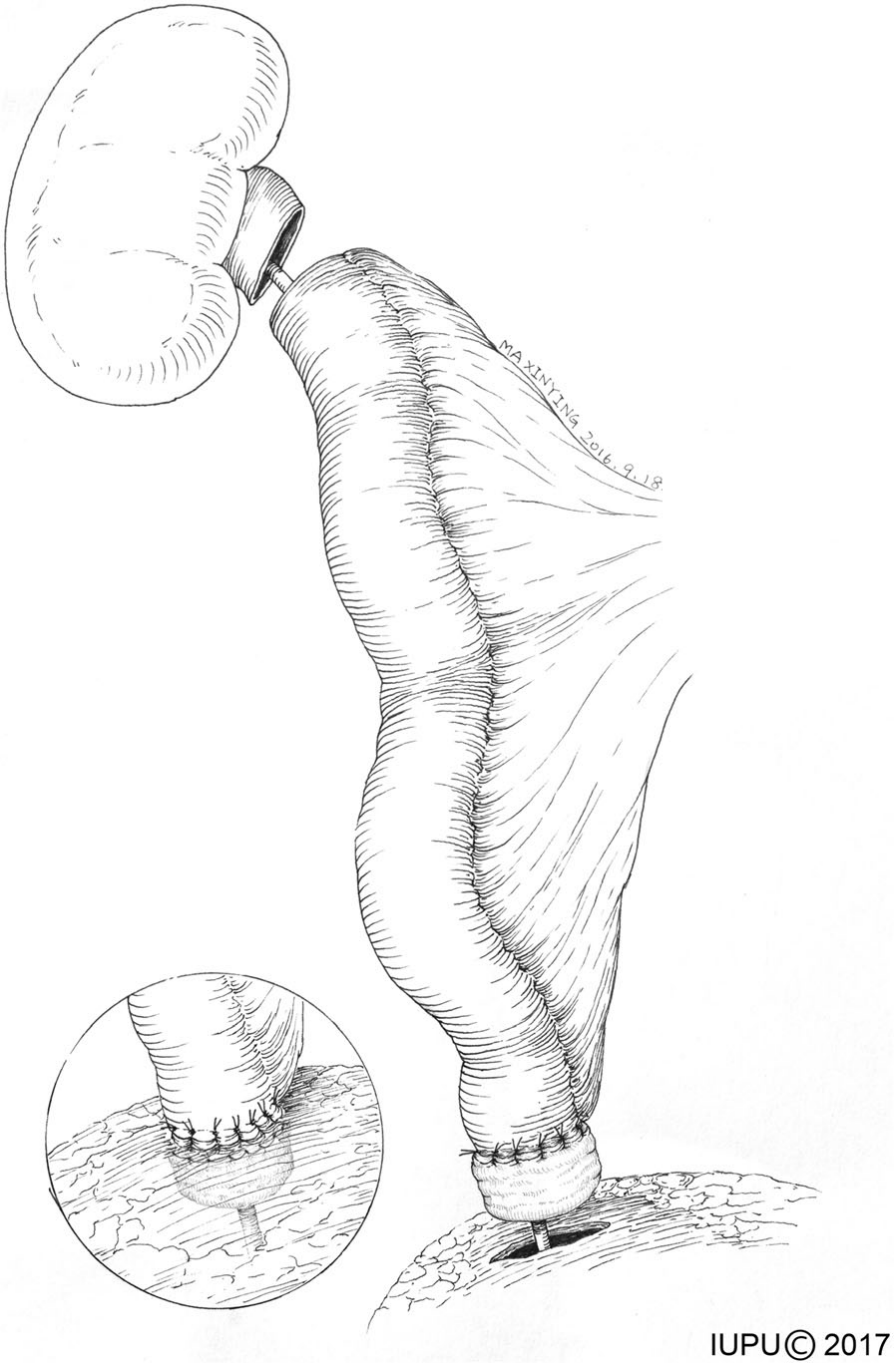
The anti-reflux nipple valve for ileal ureter substitution

**Fig. 3:**
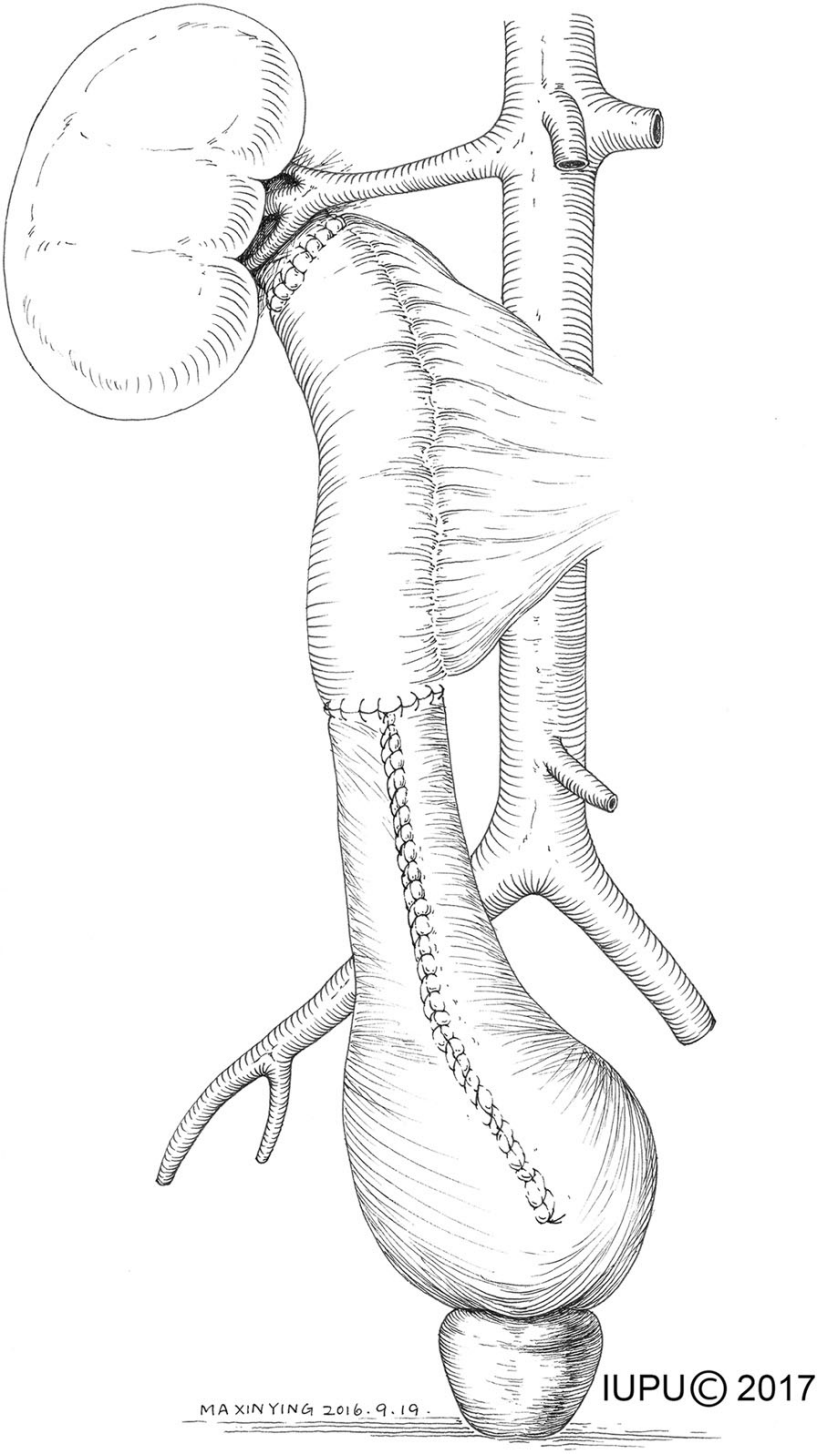
The combination of ileal ureter replacement and Boari flap-psoas hitch technique

A 6–8 F ureter stent was inserted into the isolated ileal segment. The ureter stent was fixed to the proximal and distal ends of an ileal graft to prevent dislocation. Pyeloileal and ureteroileal anastomoses were performed in an end-to-end fashion. For the bilateral ureteral replacement, a reverse “7” shaped reconstruction was performed, with the 2 proximal anastomoses on the same ileal graft. In most cases, a distal anti-reflux nipple valve was created (Fig. 2). Ileocystostomy was performed in a two-layer fashion with a running mucosa-to-mucosa suture and interrupted seromuscular–detrusor muscle suture. For the patients with preoperative borderline renal function (defined as 1.5–2.0 mg/dl), a bladder flap measuring 4 cm wide at the apex and 6–8 cm wide at the base was created, and the vesico–psoas hitch technique was performed (Fig. 3). The distal ileum with an anti-reflux nipple was anastomosed to the bladder flap. Two suction drains were placed near the proximal and distal anastomoses, and a 20F indwelling Foley catheter was inserted into the bladder.

### Follow-up

Patient follow-ups were conducted 1 and 6 months after surgery and at least once a year thereafter. The patients routinely received physical examination, blood test (including blood gas analysis, serum creatinine test, and electrolyte test), urine routine test, and radiographic examination (abdominal ultrasonography, computed tomography urography/magnetic resonance urography, and voiding cystogram) at each visit. Antegrade nephrostogram was performed postoperatively on the patient with a nephrostomy tube. The length of follow-up was defined as the interval from surgery to the last visit.

## Results

A total of 23 patients, including 13 males and 10 females, underwent ileal ureter replacement by the same surgeon between January 2010 and January 2015. The patients’ characteristics are shown in Additional file 1: Table S1. Mean age was 37.1 y (range: 16–63). The main indication for ureteral replacement in the current series was the presence of iatrogenic injuries in 15 patients (65.3%). Of these 15 patients, 11 (47.8%) had undergone urological procedures, including endoscopic ureterolithotomy in 9 patients (39.1%) and ureteral reimplantation in 2 patients (8.7%). The remaining indications are listed in Table 1. Notably, 3 patients had a solitary kidney.

**Table 1:** Indication for ileal ureter replacement

Etiology	*N* (%)
Iatrogenic injury	15 (65.3)
Urologic surgery	11 (47.8)
URSL	9 (39.1)
Others	2 (8.7)
Gynecologic surgery	2 (8.7)
Orthopedic surgery	1 (4.3)
General surgery	1 (4.3)
Car crash	2 (8.7)
Congenital obstruction	2 (8.7)
Fibrosis after radiotherapy	2 (8.7)
Tuberculosis	1 (4.3)
Ureteral TCC	1(4.3)

*URSL* ureteroscopic lithotripsy; *TCC* transitional cell carcinoma

**Fig. 4:**
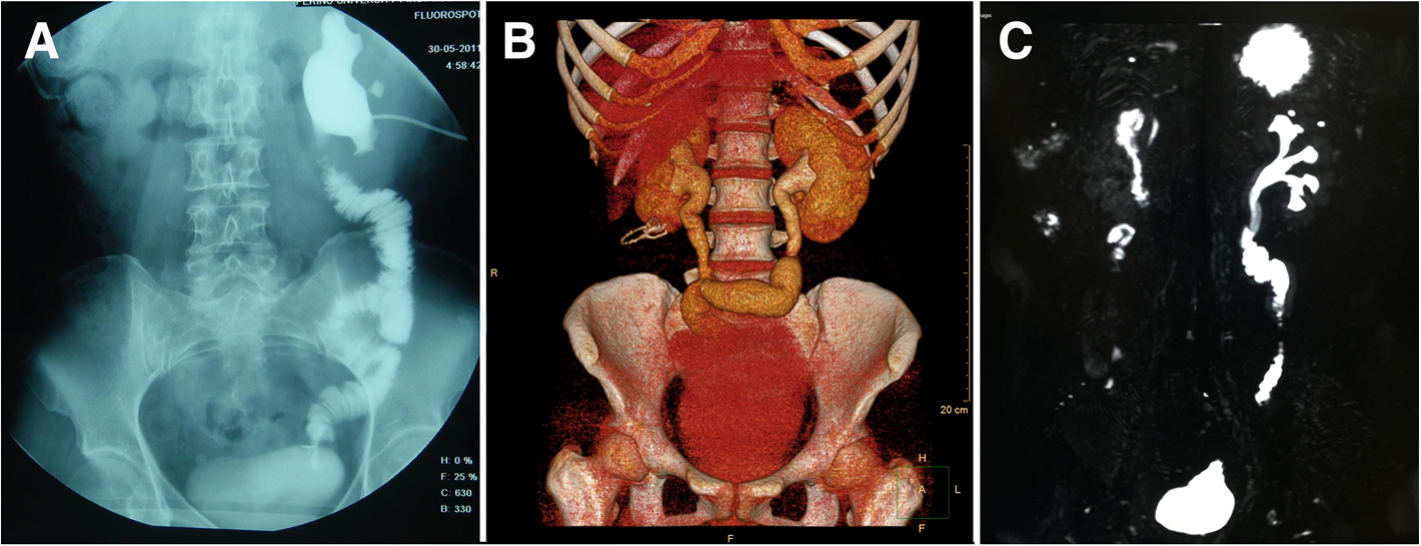
Different types of ureteral replacement. **a** unilateral ileal ureter replacement, **b**. bilateral ileal ureter replacement, **c**. combined ileal–ureter substitution and Boari flap–psoas hitch

Among the 23 patients, 8 developed ureteral injuries in the proximal mid-ureter, 2 in the mid-ureter, 8 in the distal or mid-distal ureter, and 5 in the full-length. The mean length of the injury segment was 18.6 cm (range: 5–30). All patients underwent nephrostomy prior to surgery, with a mean time of 5.5 months (range: 2–15). Among the 23 patients, 20 patients received a unilateral ileal ureter replacement, 2 received combined ileal ureter substitution and Boari flap–psoas hitch, and 1 received bilateral ileal ureter replacement. Each type of ureteral replacement is shown in Fig. 4. An anti-reflux nipple valve was created during ileocystostomy in 21 patients, and only 2 patients did not use the anti-reflux design because of the retained stone in the urinary tract. One patient required blood transfusion owing to severe adhesion around the lesion, which resulted in more blood loss (1000 mL). The median length of postoperative hospital stay was 15.1 d (range: 5–35).

With regard to the postoperative complications (Table 2), 6 minor complications (grade 2) according to the Clavien–Dindo classification developed within 30 d after surgery [10]. Urinary infection was identified as the most common complication, with 3 patients (13.0%) suffering from urinary infection. A small bowel-related complication occurred in only 1 patient with incomplete ileus, which was resolved by conservative treatment. Two patients presented incisional hernia and underwent hernia repair surgery. Only 1 patient was found to have metabolic acidosis treated with a long-term application of oral sodium bicarbonate.

**Table 2:** Postoperative complications

Complication	Patients (n)	Therapy
Early postoperative complications		
Urinary infection (Grade 2)	3	Antibiotics
ureteroileal anastomosis leakage (Grade 2)	2	Open the percutaneous nephrostomy tube for draining
Incomplete ileus (Grade 2)	1	Conservative therapy, including fast, decompression, anti-infection and balance of electrolytes.
Late postoperative complications		
Recurrent urinary infection	2	Antibiotics and sodium bicarbonate
Incisional hernia	2	Surgical repair
Metabolic acidosis	1	Oral sodium bicarbonate replacement
Stone formation	1	ESWL on ileal ureteral replacement side

*ESWL* extracorporeal shock wave lithotripsy

During a mean follow-up period of 45 months, no postoperative reflux was reported. The average creatinine levels before surgery, 1 month after surgery, and at the last follow-up visit were 1.18 (0.79–1.92), 1.07 (0.57–2.60), and 1.03 (0.76–1.69) mg/dL, respectively. Renal function in 22 patients increased or remained stable. Only 1 patient with a solitary kidney, who subsequently developed urinary infection, experienced renal deterioration after surgery. The infection was successfully managed with oral antibiotics, and the serum creatinine decreased to the normal level without major sequelae.

## Discussion

The surgical principle in managing long ureteral injury is to construct a non-refluxing and non-obstructive urinary outflow as soon as possible, thereby restoring or stabilizing the renal function. As we know, several options have been used for the reconstruction of long ureteral defects, including transureteroureterostomy, renal autotransplantation, combined Boari flap and psoa hitch, and ileal ureteral replacement. However, ileal interposition is occasionally utilized as the last resort for long ureteral obstructed segment.

Long segment ureteral defects can be caused by multiple insults, such as iatrogenic trauma, urinary tuberculosis, bilharziasis, recurrent renal calculi, retroperitoneal fibrosis, and so on [11]. The etiology of ureteral defects in the present series, compared with the earlier series, has evolved considerably. Armatys et al. [11] reported on 91 patients who underwent ureteroileoplasty. The main indication for ureteral replacement was iatrogenic injury involving genitourinary surgery in 29 cases (31.9%), nonurological surgery in 16 cases (17.6%), and radiation induced stricture in 17 cases (18.7%). Romero et al. [12] demonstrated that the most common procedure associated with iatrogenic ureteral injury was ureteroscopic stone removal. Similarly, in our study, iatrogenic injuries caused by endourological procedures, such as ureteroscopic lithotripsy, have become the main cause of long segment ureteral defect.

Contemporary series have shown promising results after prudent selection of patients in ileal interposition. Renal insufficiency before ileal interpositon is generally understood to increase the risk of metabolic acidosis. Several previous studies have emphasized that preoperative renal function is a crucial prognostic factor for ileal interposition [5, 6, 13–15]. In 1979, Boxer reported on their single-institution experience with ileal interpositon, advocating for the first time that patients with serum creatinine concentration higher than 2 mg/dL should undergo ureteral replacement cautiously [5]. Chung et al. [13] indicated that 50% of patients with serum creatinine > 2.0 mg/dL developed worsening azotemia after ureteral replacement. Wolff et al. [15] further showed a success rate > 90% when focused on patients with preoperative serum creatinine < 1.7 mg/dL.

We speculated that preoperative renal function evaluation, patient selection, and preparation are indispensable for this procedure. In our own series, after receiving internal ureteral stent or nephrostomy, patients with significant renal insufficiency were excluded. In addition, patients with borderline renal function for the procedure underwent combined ileal replacement and Boari flap–psoas hitch to minimize the length of an ileal graft. Consequently, 22 patients (95.7%) had their renal function preserved after the procedure. Metabolic acidosis was reported in only 1 patient, who underwent bilateral ureteral replacement with an ileal graft longer than 30 cm.

Several published studies have reported intestine-related complications after surgery [11, 15]. Re-establishment of small-bowel continuity presents a technical challenge to an unskilled urologist. We illustrated a simple and direct side-to-side anastomosis with the use of 2 Endo-GIA stapler loads. Small-bowel reconstruction using our technique took 3–5 min to complete. Furthermore, the technique achieved good hemostasis and reduced the risk of anastomotic stricture and leakage. Among the 23 patients, few patients reported small bowel-related complications, except for one patient who had an episode of ileus, which was resolved by conservative treatment.

There have been concerns regarding postoperative reflux. Tanagho et al. [16] reported on 5 patients who experienced progressive renal deterioration after persistent reflux. The study suggested that refluxing uropathy resulted from excessively high ileal pressure, which could lead to metabolic derangements and renal impairment. Several studies have shown that anti-reflux surgery can effectively prevent progressive loss of renal function [17–20]; regardless, whether an antireflux technique is necessary remains undetermined. Verduyckt et al. [4] compared refluxing and anti-reflux procedures in a retrospective study, concluding that the reflux rate between the two methods only slightly differed. Waldnerd et al. [21] postulated that ileal peristalsis could provide a dampening effect to retrograde reflux, and antireflux procedures were not always necessary given an ileal graft of sufficient length; they recommended the use of ≥15 cm of ileum to prevent the reflux from reaching the renal pelvis.

In our patient cohort, we used a distal anti-reflux nipple valve for the antireflux procedure in 21 patients. The remaining 2 patients with urinary stone underwent refluxing anastomosis to facilitate the expulsion of the renal stone. No cases of postoperative reflux were reported, as determined by a voiding cystogram. Despite the occurrence of potential complications, such as stenosis, desussception, and stone formation secondary to the antireflux technique [22], the creation of an inverted nipple in patients without renal stones is recommended.

Compared with the results in other recent series (Table 3) [4, 11, 13–15], those of the present series seem encouraging. On the basis of our experience, the following recommendations with respect to surgical technique are presented: (1) The ureter should be carefully isolated to diminish the risk of damage to the blood supply. Considering that exposure of the injured ureter could be difficult, in some cases with heavy inflammatory adhesion, we used a metal probe and inserted it through the nephrostomy tube to identify the position of the renal pelvis. (2) As long as a tension-free and water-tight anastomosis could be ensured, minimizing the length of ileal graft is crucial. (We divided an extra 5 cm of ileum for replacement). However, for patients with improved renal function, combined ileal interposition and Boari flap–psoas hitch may be an option. (3) Side-to-side anastomosis can increase the ease and reduce the intestine-related complications during small-bowel reconstruction. (4) The ileal segment should be maintained in an isoperistaltic orientation; alternatively, a distal antireflux procedure may be performed to prevent postoperative reflux. However, a proximal antireflux technique is unnecessary because the outflow resistance of the ureter should be eliminated before anastomosing. (5) Appropriate stenting in the ileal segment is crucial to prevent anastomotic leakage. The ureter stent should be fixed to the proximal and distal ends of the ileal graft to prevent dislocation.

**Table 3:** Outcomes of this series compared with other studies

Authors (year)	Patients (n)	Antireflux/reflux technique	Follow-up (months)	Renal function improved or stable(%)	Minor complications (%)	Major complications (%)
Verduyckt et al. [13] (2002)	18	7/11	65^a^	88.8	77.8	50
Matlaga et al. [1] (2003)	16	0/16	18.6^a^	100	16.7	0
Chung et al. [12] (2006)	56	0/56	72^a^	89.5	17.9	10.5
Armatys et al. [10] (2009)	91	2/89	36^a^	74.7	60.4	82.4
Wolff et al. [14] (2011)	17	0/17	174^b^	64.7	82.3	58.8
Present series	23	21/2	45^a^	95.7	43.5	8.7

^a^Mean

^b^Median

With the advance of laparoscopic surgery, more and more cases of laparoscopic ileal ureteric replacement have been performed in recent decades [23–27]. Stein et al. [25] presented a review of 7 patients undergoing laparoscopic interposition and 7 patients undergoing open ileal interposition. The comparative study demonstrated a significant benefit in narcotic requirement and convalescence for the laparoscopy group. In 2008, Wagner et al. [27] reported on the first case of robot-assisted laparoscopic ileal ureter, proving the feasibility of the procedure in a robotic technique. However, no studies have been reported on either laparoscopic or robotic techniques in ileal ureter replacement in China.

## Conclusion

With increasing utilization of endoscopic technology, iatrogenic long segment ureteral injury has become the main indication for ileal ureteral replacement. Despite the difficult and risky process of ileal ureter, the procedure can be carried out effectively and safely by skilled surgeons after careful patient selection.

## Additional file

Additional file 1: **Table S1.** Patients’ characteristics. (DOCX 20 kb)

## Abbreviations

CTU: Computed tomography urography
ESWL: Extracorporeal shock wave lithotripsy
F: Female
M: Male
MRU: Magnetic resonance urography
TCC: Transitional cell carcinoma
URSL: Ureteroscopic lithotripsy
