# Supplementary material for: Technical considerations and outcomes for ileal ureter replacement: a retrospective study in China
Source: BMC Surg. 2019 Jan 18;19:9. doi: 10.1186/s12893-019-0472-1 (PMC6339271; doi:10.1186/s12893-019-0472-1)
Supplement: Supplementary file 1 — Table S1. Patients’ characteristics. (DOCX 20 kb) [file 12893_2019_472_MOESM1_ESM.docx]

| Supplementary Table 1. Patients’ characteristics. | | | | | | | | |
| --- | --- | --- | --- | --- | --- | --- | --- | --- |
| Patients  no. | Sex | Age range at surgery, years | Etiology | Side of injuried segment | Site of injuried segment | Length of injuried segment, cm | Surgical procedures | Antireflux procedure |
| 1 | F | 16-63 | Urologic surgery | Left | Proximal mid-ureter | 22 | Unilateral ileal ureter replacement | No |
| 2 | F |  | Urologic surgery | Left | Distal or mid-distal ureter | 15 | Unilateral ileal ureter replacement | Yes |
| 3 | F |  | Gynecologic surgery | Right | Full-length ureter | 25 | Unilateral ileal ureter replacement | Yes |
| 4 | F |  | General surgery | Left | Distal or mid-distal ureter | 15 | Unilateral ileal ureter replacement | Yes |
| 5 | F |  | Urologic surgery | Right | Proximal mid-ureter | 13 | Unilateral ileal ureter replacement | Yes |
| 6 | F |  | Gynecologic surgery | Left | Distal or mid-distal ureter | 20 | Unilateral ileal ureter replacement | Yes |
| 7 | F |  | Ureteral TCC | Left | Full-length ureter | 20 | Unilateral ileal ureter replacement | Yes |
| 8 | F |  | Urologic surgery | Right | Proximal mid-ureter | 15 | Unilateral ileal ureter replacement | Yes |
| 9 | F |  | Fibrosis after radiotherapy | Right | Distal or mid-distal ureter | 15 | Unilateral ileal ureter replacement | Yes |
| 10 | F |  | Fibrosis after radiotherapy | Bilateral | Distal or mid-distal ureter | 15 | Bilateral ileal ureter replacement | Yes |
| 11 | M |  | Urologic surgery | Left | Distal or mid-distal ureter | 20 | Unilateral ileal ureter replacement | Yes |
| 12 | M |  | Urologic surgery | Left | Proximal mid-ureter | 25 | Unilateral ileal ureter replacement | Yes |
| 13 | M |  | Congenital obstruction | Right | Proximal mid-ureter | 20 | Unilateral ileal ureter replacement | Yes |
| 14 | M |  | Urologic surgery | Right | Distal or mid-distal ureter | 15 | Unilateral ileal ureter replacement | Yes |
| 15 | M |  | Car crash | Left | Proximal mid-ureter | 15 | Unilateral ileal ureter replacement | Yes |
| 16 | M |  | Congenital obstruction | Right | Proximal mid-ureter | 5 | Unilateral ileal ureter replacement | Yes |
| 17 | M |  | Orthopedic surgery | Right | Proximal mid-ureter | 15 | Unilateral ileal ureter replacement | Yes |
| 18 | M |  | Tuberculosis | Right | mid-ureter | 15 | Unilateral ileal ureter replacement | Yes |
| 19 | M |  | Urologic surgery | Right | Full-length ureter | 30 | Unilateral ileal ureter replacement | Yes |
| 20 | M |  | Urologic surgery | Right | Full-length ureter | 25 | Unilateral ileal ureter replacement | No |
| 21 | M |  | Urologic surgery | Left | Distal or mid-distal ureter | 30 | Ileal ureter replacement + Boari flap-psoas hitch | Yes |
| 22 | M |  | Car crash | Left | Full-length ureter | 20 | Ileal ureter replacement + Boari flap-psoas hitch | Yes |
| 23 | M |  | Urologic surgery | Right | mid-ureter | 18 | Unilateral ileal ureter replacement | Yes |
| F = female; M = male; TCC = transitional cell carcinoma | | | | | | | | |
